# Selection of Culture Conditions and Cell Morphology for Biocompatible Extraction of β-Carotene from *Dunaliella salina*

**DOI:** 10.3390/md19110648

**Published:** 2021-11-22

**Authors:** Guillaume Tanguy, Aline Legat, Olivier Gonçalves, Luc Marchal, Benoît Schoefs

**Affiliations:** 1 Laboratoire GEPEA, Université de Nantes, Oniris, UMR 6144, 44600 Saint-Nazaire, France; guillaume.tanguy1@etu.univ-nantes.fr (G.T.); alinelegat@outlook.fr (A.L.); olivier.goncalves@univ-nantes.fr (O.G.); luc.marchal@univ-nantes.fr (L.M.); 2 Metabolism, Bio-Engineering of Microalgal Molecules and Applications, Laboratoire Mer Molécules Santé, IUML–FR 3473 CNRS, Le Mans Université, Avenue Olivier Messiaen, 72085 Le Mans, France

**Keywords:** microalgae, *Dunaliella salina*, β-carotene, in situ extraction, milking, biocompatibility, photobioreactor

## Abstract

Biocompatible extraction emerges recently as a means to reduce costs of biotechnology processing of microalgae. In this frame, this study aimed at determining how specific culture conditions and the associated cell morphology impact the biocompatibility and the extraction yield of β-carotene from the green microalga *Dunaliella salina* using *n*-decane. The results highlight the relationship between the cell disruption yield and cell volume, the circularity and the relative abundance of naturally permeabilized cells. The disruption rate increased with both the cell volume and circularity. This was particularly obvious for volume and circularity exceeding 1500 µm^3^ and 0.7, respectively. The extraction of β-carotene was the most biocompatible with small (600 µm^3^) and circular cells (0.7) stressed in photobioreactor (30% of carotenoids recovery with 15% cell disruption). The naturally permeabilized cells were disrupted first; the remaining cells seems to follow a gradual permeabilization process: reversibility (up to 20 s) then irreversibility and cell disruption. This opens new carotenoid production schemes based on growing robust β-carotene enriched cells to ensure biocompatible extraction.

## 1. Introduction

Microalgae have been identified as promising sources for the production of biomolecules for energy, food, feed, health, pharmaceutical, and cosmetic industries [1]. Among the biomolecules extracted from microalgae, carotenoids occupy a historical and prominent place [2] because they exhibit unique properties such as provitamin A, antioxidant and skin protection activities [3]. The powerful antioxidative effect of carotenoids is due to the scavenging of free radicals. Its positive effect on health has been proven in the treatment of a wide number of diseases with antidiabetic, antitumor, and anti-inflammatory activities and benefits for cognitive function [3].

β-carotene is an ubiquitous pigment in microalgae due to its requirement for the photosynthetic process [2]. In a few taxa, such as the green microalga *Dunaliella* sp., the coupled application of nitrogen depletion and high light stress triggers the reorientation of the carbon metabolism toward the massive accumulation of β-carotene: up to 14% of dry weight (DW) [4]. Current microalgal fractionation processes require several downstream steps such as dewatering, drying, solvent extraction and purification of the target biomolecules [5]. In addition, they generate residues that need to be treated. Most of these steps consume time and energy intensively [6], which jeopardizes the ecology and economic viabilities of these processes unless the prices of this type of biomolecules are very high. The expensive market prices of those active compounds from microalgae ultimately reduce their uses for medical treatment and favour the use of chemically synthetized molecules [2]. To reduce production residues, in situ extraction methods or milking have been proposed. This can include the use of pre-treatments such as electric pulses [7,8], resonance frequency [9], or biocompatible solvents [10,11,12,13,14]. In *Dunaliella salina*, by comparing β-carotene solubility, solvent extraction ability and biocompatibility of organic solvents, León et al. (2003) established that the best compromised for β-carotene biocompatible extraction was achieved with hydrophobic solvents exhibiting a log P_octanol_ around five such as *n*-decane, provided that the solvent/culture ratio (*v*/*v*) remains less than 1 [12,13]. Nevertheless, for *D. salina*, *n*-decane was considered either as non-compatible [14] with a direct correlation between cell destruction and β-carotene extraction [15], or biocompatible [13] with proportionally more β-carotene extracted than cells disrupted. It has also been reported that lipid extraction from microalgae with organic solvent depends on the contact time and area between the culture media and the organic solvent, but the higher the extraction the lower the biocompatibility [11,14,16,17,18].

While many studies focused on solvent choice and extraction operating conditions, there is a lack of understanding of the impact of culture conditions on the single cell characteristics and de facto on the extraction mechanism(s). *D. salina* is a wall-less microalga, i.e., the cells are limited by a thin and elastic plasma membrane [18] that acts as the sole selective barrier to compounds [19]. As an assumption, the extraction can only be biocompatible for a cell if any possible alteration of the membrane is reversible. Biological membranes are the preferential places where hydrophobic solvents such as *n*-alkanes can accumulate [20], disturb the membrane fluidity [21] and eventually lead to its permeabilization [22].

The influence of *D. salina* morphology on the β-carotene extraction with *n*-decane as well as cell preservation during the process were investigated using a multidisciplinary approach including cell biology, biochemistry, physiology and processing. These data will be crucial for the establishment of future biotechnological processes aiming at extracting β-carotene from microalgae.

## 2. Results

### 2.1. β-Carotene Extraction from Cells

**Figure 1:**
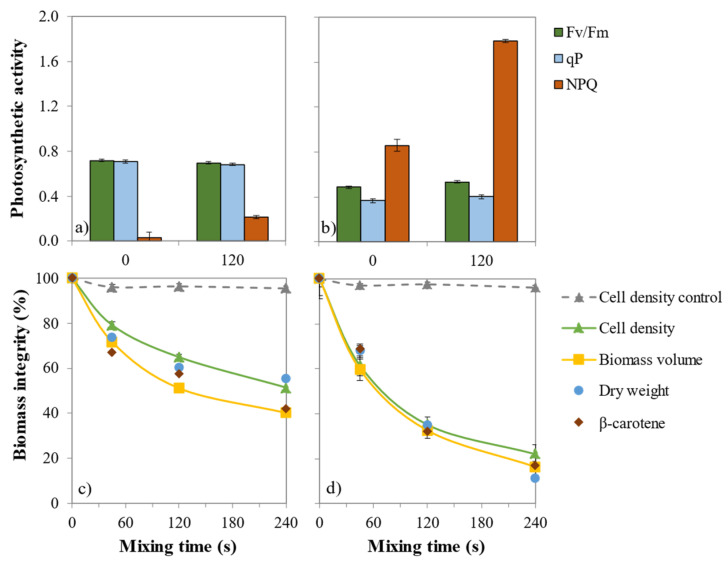
Biocompatibility of *n*-decane treatment. Photosynthetic activity (**a**,**b**) before stirring (0 s) and after 120 s mixing time for (**a**) grown cells (GE) and (**b**) stressed cells (SE). Biomass integrity (%) (**c**,**d**) expressed as cell density (control: stirring without solvent), biomass volume, dry weight and β-carotene content for GE (**c**) and SE (**d**) cells with solvent mixing time. Scale bars indicate standard deviation error (*n* = 3).

The ability of *n*-decane to extract β-carotene from cells of *D. salina* Grown in Erlenmeyer (GE) and Stressed in Erlenemeyer (SE) was determined through the establishment of the extraction kinetic (Figure 1c,d). GE and SE cells initially have a β-carotene content of 4 and 38 pg cell^−1^, respectively. Regardless the cell stress state, the β-carotene extraction followed a first-order kinetic with up to 60% recovery in *n*-decane in 240 s for GE and 88% for SE. The extraction rate was twice higher for SE cells than for GE cells (k_SE_ = 7.3 × 10^−3^ s^−1^, k_GE_ = 3.3 × 10^−3^ s^−1^, in the same mixing-settling conditions indicating a pigment extraction mechanism with lower resistance when β-carotene was over-accumulated. A 90% extraction yield was then expected to be obtained in 315 and 700 s for SE and GE, respectively.

### 2.2. Impact of β-Carotene Extraction on Photosynthetic Activity

Before solvent treatment, the maximum quantum yield of photosystem II photochemistry (Fv/Fm) reached 0.7 values typical of GE cells (Figure 1a,b). Under the irradiance used, most of the absorbed photons are used for photochemistry as shown by the high value of photochemical quenching (qP) and therefore, the need to dissipate excessive photons is very reduced as shown by the low value of non-photochemical quenching (NPQ). The values of these parameters are contrasted in SE cells (Figure 1b). Fv/Fm value of 0.5 indicates that photosystem II reaction centres have been damaged and that their ability to use the energy associated with the absorbed photons is reduced. Altogether, these results fit with the dismantling of the photosynthetic membranes [4] as reflected by the reduction of the photosynthetic oxygen emission capacity [23] and of Fv/Fm [24] during β-carotene accumulation. Therefore, the need to dissipate this excess of energy as heat using an NPQ mechanism is strongly increased. This increase is tremendously larger after mixing with *n*-decane (Figure 1b), probably due to the decreased light-filtering effect exerted by the accumulated chloroplastic β-carotene lipid droplets. The solvent treatment partly extracted the β -carotene, and the amount of photons reaching the light-harvesting antenna complexes is higher, increasing the photon excitation pressure (1-qP), [25]. The absence of a decrease in Fv/Fm after 120 s of mixing indicates that the solvent did not significantly impair the photosynthetic machinery. Our results confirm the low toxicity of *n*-decane on the photosynthetic activity of GE and SE cells was reached by several other studies (e.g., [12,14]).

### 2.3. Impact of β-Carotene Extraction on Cell Integrity

To evaluate the effect of the *n*-decane extraction on GE and SE cells integrity, β-carotene content, dry weight, cell density and biomass volumes were measured throughout the treatment. First, as a control sample, for GE and SE cells, there was no significant cell disruption (<3%) using vortex treatment alone (Figure 1c,d). Then for GE, the comparison of the β-carotene release and cell density decrease kinetics (65% of remaining β-carotene and 78% of the intact cells at 45 s) indicates that a part of carotenoids are extracted without any cell disruption or that there is a possible heterogeneity in cell content (disrupted cells contain more carotenoids than intact ones) or in the cell disruption (cells that contain more carotenoids are preferentially disrupted).

For SE cells, the β-carotene release and cell density decrease kinetics are similar (83% extracted β-carotene and 78% cell disruption at 120 s), indicating in this case that extraction and disruption are directly correlated or that the culture content is homogeneous.

For GE cells, after 240 s of mixing, the biomass volume had a 60% decrease while the cell density decrease was only 49% (Figure 1c). The difference was less pronounced for SE cells: 88% biomass volume decrease and 80% cell density decrease (Figure 1b). The decrease in biomass dry weight and volume were similar; no cell shrinkage occurred during extraction. In both culture conditions, after 120 s of mixing, the higher decrease in volume was related to the higher extraction yield. The decrease in average cell volume of *D. salina* cultured in the presence of *n*-dodecane was previously reported by Kleinegris et al. (2011a) and this behaviour extends the correlation between β-carotene extraction and cell death established by Kleinegris et al. (2011b) to cell disruption as a prerequisite for β-carotene extraction [15,26]. Because larger cells have higher carotenoids content [27] the most likely hypothesis to explain GE behaviour is that they are preferentially disrupted during the liquid–liquid extraction with *n*-decane.

### 2.4. Cell Size Distribution

**Figure 2:**
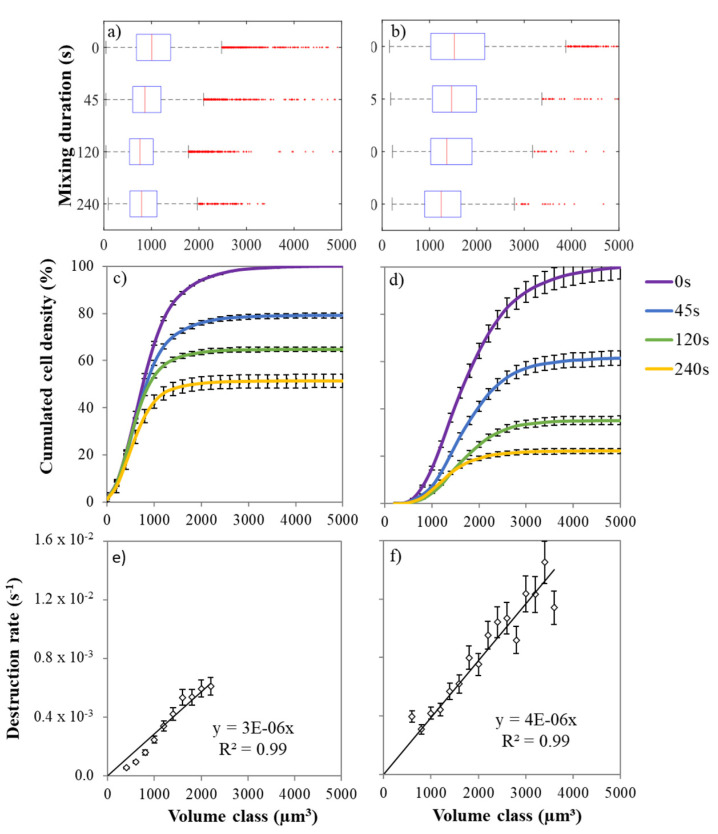
Cell volume-dependent disruption for (GE) grown cells (**a**,**c**,**e**) and (SE) stressed cells (**b**,**d**,**f**). Box plot of the distribution (**a**,**b**). Cumulated cell density per volume class (**c**,**d**). Cell disruption rate by volume class (µm^3^) (**e**,**f**). Scale bars indicate standard deviation (*n* = 3). Median and third quartile of the distribution were more affected than first quartile (*p* < 0.05).

The cell size distribution by volume classes does not follow a normal distribution [28] (Figure 2a,b). The skewness is due to the presence of a higher number of cells with volumes larger than the median i.e., 1000 and 1500 µm^3^ for GE and SE cultures, respectively. As shown by the quantile values, the population heterogeneity was larger in SE culture than in GE culture. Regardless the stress state, contact with *n*-decane reduced the number of cells in each individual cell volume classes (Figure 2c,d). The larger cells were the most affected, i.e., those above the median and third quartile of the distribution (*p* < 0.05). For each volume class, cell disruption rate was followed and modelled by a first-order kinetic function with a k_d_ parameter (s^−1^). For the GE cells, disruption kinetics for each volume class increased linearly (R^2^ > 0.99) from k_d_ = 5.4 × 10^−4^ s^−1^ for the 200 µm^3^ class to k_d_ = 5.9 × 10^−3^ s^−1^ for the 2200 µm^3^ class (Figure 2e). Values for higher cell volumes are not calculated because of insufficient data. Large *D. salina* cells were then disrupted ten time faster than the small ones, confirming the heterogeneity hypothesis of the previous section. For SE cells, the *D. salina* cells were bigger with a wider cell size distribution (Figure 2a,b at 0 s). The disruption rate by volume class also increased linearly (R^2^ > 0.99) according to the cell size (Figure 2f) up to k_d_ = 1.4 × 10^−2^ s^−1^ for 3500 µm^3^ cells.

### 2.5. Influence of Volume and Circularity on Cell Weakness

**Figure 3:**
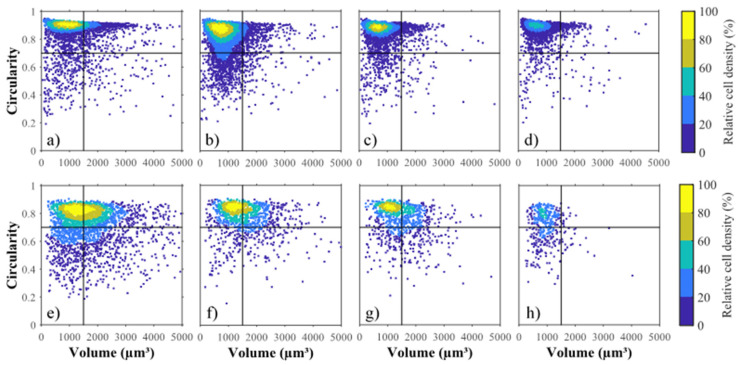
Effect of *n*-decane extraction on circularity and volume. Density scattering plots of grown cells (GE), (**a**–**d**) for 0 s (**a**), 45 s (**b**), 120 s (**c**) and 240 s (**d**) and stressed cells (SE), (**e**–**h**) for 0 s (**e**), 45 s (**f**), 120 s (**g**) and 240 s (**h**).

**Figure 4:**
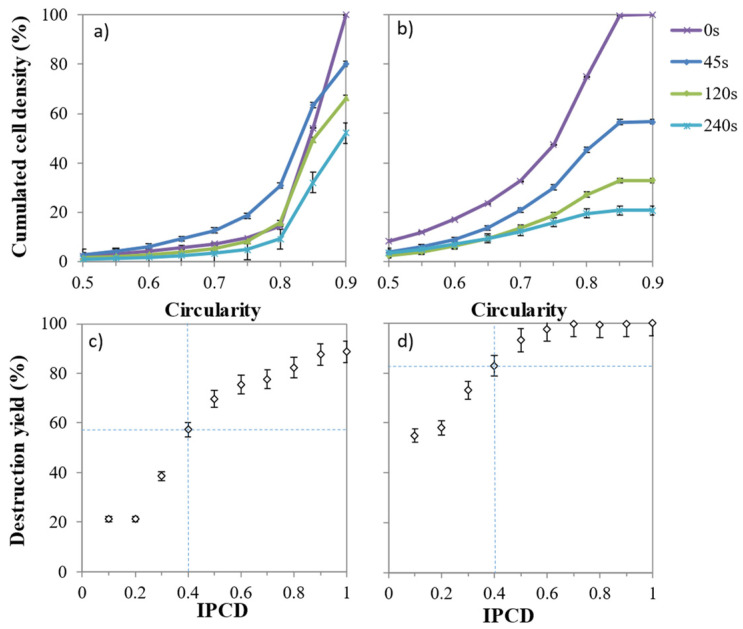
The index of potential cell disruption. Cumulated cell density per circularity class (**a**,**b**) for (**a**) grown cells (GE) and (**b**) stressed cells (SE). Disruption yield (%) per index of potential disruption class (IPCD), (**c**,**d**) for GE cells (**c**) and SE cells (**d**). Scale bars indicate standard deviation (*n* = 3). The higher the IPCD the higher the disruption yield (*p* < 0.05).

The density scatter plots displayed in Figure 3a–h show the distribution of cell volume and cell circularity throughout the liquid–liquid extraction with *n*-decane. As expected, GE cells are mainly characterized by high circularity, ranging in 0.85–0.95 and with an initial mean cell volume around 1000 µm^3^ (Figure 3a). After 45 s of mixing with the solvent, 15% of cells dropped in circularity between 0.60 and 0.80 (Figure 3b and Figure 4a) while the cells that initially had a cell volume greater than 1500 µm^3^ decreased significantly. Low-circularity cells progressively disappeared (Figure 3c,d). The transient decrease in circularity for short contact time with the solvent (less than 45 s) induced a cell distortion, which may be due to a cell membrane alteration as confirmed by electron microscope observations by Hejazi et al. (2004) and Li et al. (2015) [29,30]. Cells that survived to longer exposure (up to 240 s) to *n*-decane were few, small and showed circularity ranging between 0.70–0.90.

As shown by the light blue contour (Figure 3e), the 80% core population of stressed cells is distributed in a more diffuse pattern in terms of circularity and volume. All categories of cells were altered by the solvent treatment, but those characterized by a circularity less than 0.50 or cell volume greater than 1500 µm^3^ were almost absent after 240 s. Unlike for GE cells, the transient decrease in single cell circularity was not observed for SE cells (Figure 4b).

The volume and circularity criteria were unified with an index of potential cell disruption (IPCD) (Equation (8)), that varies between 0 and 1, to model the overall weakness. It was calculated as the product of normalized volume (Equation (6)) by the normalized circularity (Equation (7)) (Section Biomass Integrity, Cell Disruption Yield and Rate). The higher the IPCD, the higher the disruption yield (*p* < 0.05) (Figure 4). Similar sigmoidal curves with inflection point around IPCD = 0.4 were found. They differed, however in the initial relative destruction; it was much higher for SE than for GE ones, suggesting that there are other biological parameters that influence the cell disruption.

### 2.6. Biocompatible Extraction from Photobioreactor Production

**Figure 5:**
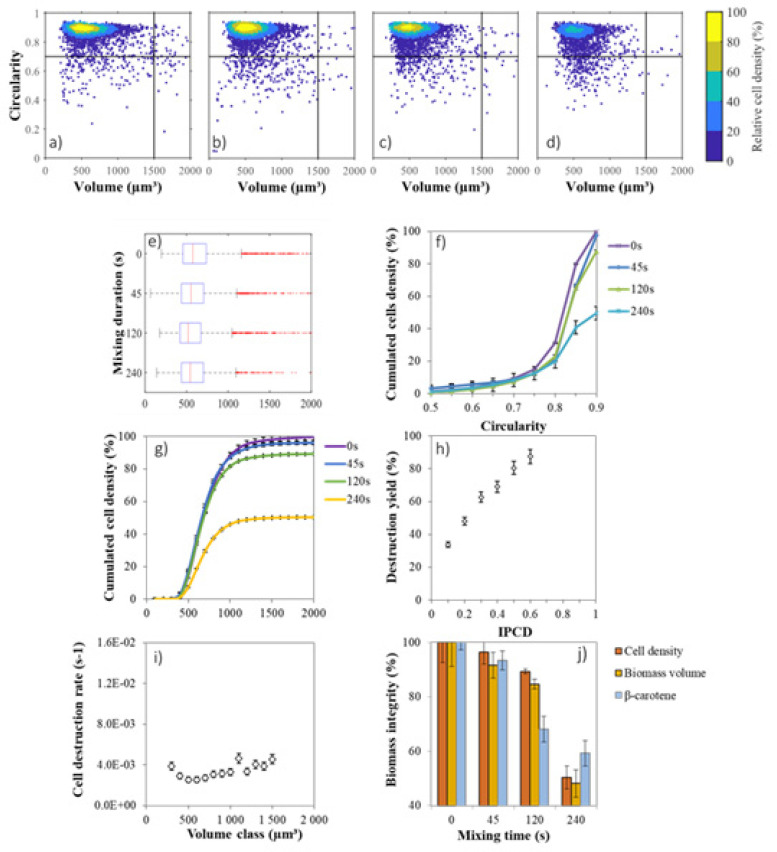
Extraction from robust cells stressed in photobioreactor (SPBR). Density scattering plots (**a**–**d**) for 0 s (**a**), 45 s (**b**), 120 s (**c**) and 240 s (**d**). Box plot of the distribution (**e**). Cumulated cell density per circularity class (**f**). Cumulated cell density per volume class (**g**). Disruption yield (%) per index of potential cell disruption (IPCD) (**h**). Cell disruption rate by volume class (µm3), (**i**). Biomass integrity of cells with mixing duration (**j**), biocompatible extraction is the most apparent at 120 s mixing. Scale bars indicate standard deviation (*n* = 3).

The improved design of photobioreactors allows us to culture microalgae under more controlled conditions such as pH, mixing and CO_2_ supply to increase their growth rate [31]. The density scatter plot displayed in Figure 5a shows the distribution of cell volume and cell circularity for the stress in photobioreactor (SPBR) cells. The applied culture conditions led to more homogeneous cell characteristics than in erlenmeyer with about 0.9 in circularity (Figure 5f) and 600 µm^3^ mean cell volume (Figure 5e), which was three times smaller than for SE cells. Interestingly however, SPBR cells had 20% less intracellular β-carotene. Because SPBR cells received less light energy (18 µmol g_DW_^−1^ s^−1^) than SE cells (42 µmol g_DW_^−1^ s^−1^), their volumes were smaller as shown by Xu et al. (2016) [32].

After 120 s of solvent extraction the cell population decreased by 10% (Figure 5g) and only 2% of cells dropped to 0.40–0.60 in circularity (Figure 5f). Then at 240 s, the cell density decreased to 45% and only 2% of cells decreased in 0.50–0.70 in circularity. The cell volume-dependent disruption rate was not apparent with these batch culture conditions (Figure 5i).

The IPCD for SPBR and GE cells were similar (Figure 5h) but the destruction rate by IPCD was more linear for SPBR. At IPCD = 0.4, the disruption rate was 60, 80 and 70% for GE, SE and SPBR, respectively. This indicates that apart from the difference in volume and circularity, SPBR cells were slightly more robust than SE cells. Small cells (around 600 µm^3^) with a circular membrane (around 0.9 in circularity) were able to maintain their membrane integrity for extraction time up to 120 s while 240 s extraction led to a loss in cell density and biomass volume (50%).

While a slight biocompatible extraction seemed to be possible at 120 s (Figure 5j), (30% of carotenoids recovery with 15% cell disruption) an opposite result appeared at 240 s (40% of carotenoids recovery with 50% cell disruption).

### 2.7. Cell Permeabilization with Solvent

**Figure 6:**
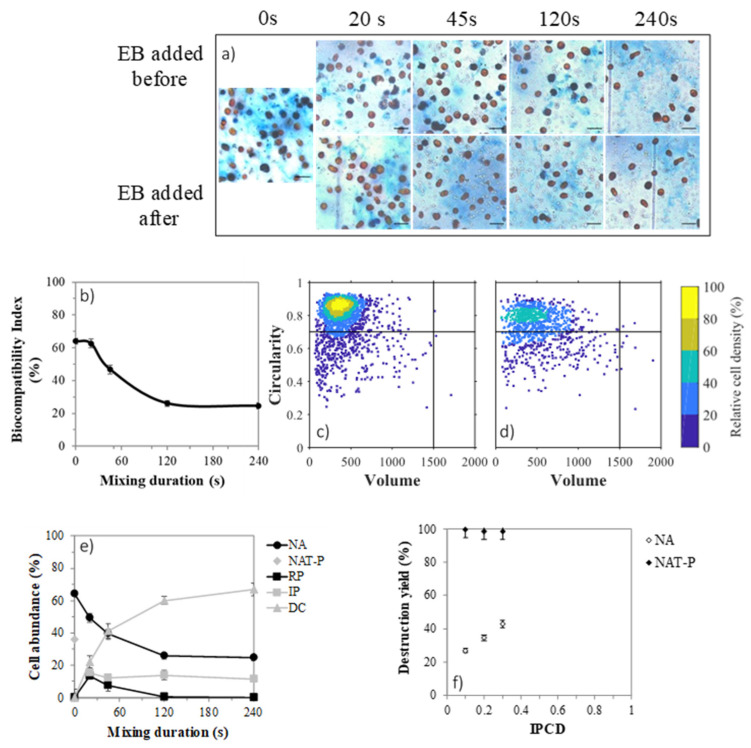
Membrane permeabilization with *n*-decane of SPBR+E cells. Pictures of the different permeabilization categories of cells (**a**) black line represents 20 µm. Cell abundance (%) in disrupted cells (DC), non-affected cells (NA), irreversibly permeabilized cells (IP) and reversibly permeabilized cells (RP) for the different extraction time (**b**). Biocompatibility index throughout the treatment (**c**). Density scatter plots of non-affected cells (**d**) and naturally permeabilized cells (**e**). Cell disruption yield per index of potential cell disruption (IPCD) (**f**).

To highlight the kinetic of permeabilization and understand the higher IPCD values of SE compared to GE and SPBR culture conditions, the mixing of *D. salina* with *n*-decane was carried out with SPBR cells then cultured in erlenmeyer (SPBR+E) to increase the abundance of fragile cells as in the SE cell state. Cells were stained with Evans blue (EB) to measure the percentage of permeabilized cells. A representative picture of samples with EB added before or after extraction is shown in Figure 6a. In the absence of contact with the solvent, many cells loaded EB and appeared dark in colour, meaning that naturally permeabilized (NAT-P) subpopulation accounted for 36% of the microalgae tested (Figure 6e). This proportion is similar to that reported in the literature (Gateau et al., 2021) [8]. Among the NAT-P subpopulation, some cells presented an irregular membrane and were partially opened (Figure 6a).

As seen with the colour contour, the core population of NAT-P cells (Figure 6d) overlap the one of non-affected (NA) (Figure 6c), thus on average NAT-P and NA had the same volume and circularity. In consequence the natural permeabilization is independent of the circularity and the volume.

After 20 s, there was 21% of disrupted cells (DC), 15% of cells were irreversibly permeabilized (IP), 13% turned reversibly permeabilized (RP), and NA decreased from 64 to 50%. Because they are both stained when EB was added after mixing, NAT-P and IP cannot be distinguished. However, the DC (21%) corresponded to the difference between NAT-P (36%) and IP (15%) and the decrease in NA (14%) corresponded to the increase in RP (13%). Therefore, it can be hypothesized that: (i) the NAT-P were more fragile and disappeared first and (ii) NA cells were more robust and turned RP. For longer mixing time, the RP cells became progressively less abundant while that of IP remained constant (Figure 6e). Gateau et al. [8] have coined the biocompatibility index (BI) to describe the evolution of such populations during a permeabilizing treatment [8]. Figure 6b displays the evolution of BI along the *n*-decane treatment. An exposure time longer than 45 s led to a decrease in NA and BI. However, the NAT-P cells are considered as dying cells; their death will not effectively decrease the biocompatibility. Therefore, the treatment could be considered as mainly biocompatible for very short extraction time, i.e., <45 s. To summarize, NAT-P were disrupted first, and NA seemed to follow a gradual permeabilization process: RP→IP→DC. The presence of RP cells was only transient as its proportion decreased when the treatment time exceeded 20 s. The disruption yield for NAT-P cells was around 100% for all IPCD (Figure 6f).

## 3. Materials and Methods

### 3.1. Strain and Cultures in Flask

The green microalga *Dunaliella salina* CCAP 19/18 was cultivated in 500 mL glass erlenmeyer fed with 200 mL of Johnson growth medium modified according to Çelekli and Dönmez, (2006) [33]. Cultures were grown in a room with a thermostat (20 ± 2 °C) under a continuous photon flux density (35 ± 5 µmol m^−2^ s^−1^) and agitated manually once a day. The cultures were subcultured in fresh medium every four weeks to reach a cell density, a dry weight and a mean volume of circa 2.1 × 10^6^ cell mL^−1^, 0.70 g_DW_ L^−1^ and 1100 µm^3^. This culture condition is referred as grown in erlenmeyer (GE).

To trigger β-carotene accumulation, GE cells were harvested in sterile Falcon tube, centrifuged (6000× *g*, 10 °C, 10 min), resuspended in a glass erlenmeyer in the same medium but free of nitrogen (nitrogen depleted medium) and continuously illuminated from the bottom using a photon flux density (PFD) of 800 µmol m^−2^ s^−1^ provided by LED panels (neutral white, 4000–4500 K) for four weeks to reach a cell density, a dry weight and a mean volume of circa 9.7 10^5^ cell mL^−1^, 0.63 g_DW_ L^−1^ and 1700 µm^3^, respectively. Cells cultivated under these conditions are referred as stress in erlenmeyer condition (SE).

### 3.2. Culture in Photobioreactor

To produce cells in a photobioreactor (PBR), 100 mL of the GE culture were added into 1 L airlift flat panel PBR in the previous medium with doubled concentration of nitrogen, sulphur and phosphorus and maintained at pH = 7.5 and at 400 ± 35 µmol m^−^^2^ s^−1^ supplied by LED panels (neutral white, 4000–4500 K) to reach a cell density of 8.2 × 10^6^ cell mL^−1^ after one week. Then, cells were harvested and resuspended in nitrogen-free medium in PBR for one week, as described in the previous section, but the PFD was increased to 1200 µmol m^−2^ s^−1^ to reach a cell density, a dry weight and a mean volume of *circa* 1.6 × 10^6^ cell mL^−1^, 2.2 g_DW_ L^−1^ and 600 µm^3^, respectively This culture condition is referred as stress in photobioreactor (SPBR). A sample of 200 mL of SPBR culture was then harvested, transferred in an erlenmeyer without medium modification and the GE conditions were applied to allow the development under less controlled conditions; these cells are referred as SPBR+E.

The specific irradiance I_sp_ (µmol g_DW_^−1^ s^−1^) was calculated as the photon flux density PFD (µmol m^−2^ s^−1^) divided by the dry weight of the biomass DW (g_DW_ L^−1^) and multiplied by the specific illuminated area a_light_ (m^2^ m^−3^) as expressed in Equation (1) [34]:

I_sp_ = PFD/(DW × 1000) × a_light_ (1)

The specific illuminated area a_light_ can be calculated with the culture depth L using the Equation (2). The culture depth in PBR was 0.03 m. Due to the illumination from the bottom and to simplified calculations, the same equation was used for erlenmeyer; the light path was also 0.03 m.

a_light_ = 1/L (2)

### 3.3. In Situ Extraction Experiment

β-carotene was extracted in a 50 mL Falcon tube using *n*-decane (ACROS Organics, >99%). Microalga culture concentration was adjusted with fresh medium to reach 0.63–0.70 g_DW_ L^−1^ and then mixed with *n*-decane (1:1 *v*/*v*) for 45, 120 or 240 s using a vortex (Fischerbrand) vibrating at 25 Hz. The Falcon tubes were immediately centrifuged (4000× *g*, 15 °C, 10 min), which resulted in the recovery of three phases: the lower aqueous phase (residue), the upper organic phase (extract) and a stable emulsion at the interface.

For a 1st order-like extraction kinetic, concentration profile (%) can be described by a single parameter model k (s^−1^) following Equation (3):

C_b-carot_ (%) = e^−k·t^ (3)

### 3.4. Characterization of the Aqueous and Solvent Phases

#### 3.4.1. Photosynthetic Activity

Previous studies (e.g., [19]) have regularly used the light-dependent oxygen emission from the photosynthetic apparatus for evaluating the biocompatibility of solvent treatment on microalgae. However, these results might be biased as the solubility of oxygen in aqueous media is lower than in organic solvents [35]. To avoid this difficulty, the biocompatibility of the treatment with *n*-decane was studied from the microalgal physiology point of view using the variations of the chlorophyll fluorescence quantum yield, a non-destructive tool for measuring the photosynthetic activity [36]. Photosynthetic activity was measured according to Roháček et al. (2014) [37]. Maximum quantum efficiency of the photosystem II (Fv/Fm), photochemical (qP) and non-photochemical quenching (NPQ) were calculated according to Roháček et al. (2008) [36]. The maximum quantum efficiency (Fv/Fm) of the photosystem II (PSII) photochemistry quantifies the maximum photochemical efficiency of open reaction centres.

#### 3.4.2. β-Carotene Quantification in Biomass

β-carotene concentration in the biomass was measured before and after each solvent treatment time. For this purpose, 0.5 mL sample of cell culture was centrifuged (4000× *g*, 15 °C, 10 min) and the pellet was resuspended in 1.5 mL of methanol (ACROS Organics, >99%) at 44 °C for 45 min then centrifuged again and the absorbance of the supernatant was measured at 470, 653, 666 and 750 nm. The β-carotene was quantified spectrophotometrically according to the equations of Lichthentaler and Wellburn (1983) [38]. According to Mojaat et al. (2008b) the β-carotene represented the main carotenoid (>95%) [39]. Therefore, β-carotene concentration was approximated with total carotenoids concentration measured by spectrophotometry.

#### 3.4.3. Dry Mass

Five mL of *D. salina* culture were filtered on pre-dried and pre-weighted fiberglass filters (Whatman GF/F, 0.7 μm) using a Buchner funnel. To avoid overweighting due to salt crystallization; the filters were rinsed with 15 mL of isotonic ammonium formate (100 g L^−1^, ACROS Organics, >99%), [40]. The filters were then dried in oven at 110 °C for 24 h, cooled in a desiccator for 10 min and weighed (Sartorius, SECURA225D-1CEU).

#### 3.4.4. Cell Density, Volume and Circularity

##### Cell Density

Cell density, cell volume and cell circularity were determined on a Nageotte slide after cell immobilization (500:50 *v*/*v*) with a 4% solution of paraformaldehyde (PF), (ACROS Organics, Illkirch, France, >96%) in saline phosphate buffer. The treatment did not affect the morphology or the colour of the microalgae [41]. Sixty microscopy bright fields (AxioVision A1, Zeiss, Iena, Germany; magnification: ×400, picture resolution: 0.25 µm px^−1^) were pictured randomly. The colour pictures (16 bit) were transformed into B/W ones (8 bit) using the Image J software (https://imagej.nih.gov/ij/, accessed date: 9 October 2021, National Institutes of Health), which were then subjected to particle analysis using a default Image J routine. Briefly, cell debris and other small particles were first automatically removed from the analysis by setting the “cell area” parameter to the range 20–400 µm^2^ and by manual sorting. A minimum of 1600 cells were counted, limiting the standard error of cell concentration estimation to 5% [42]. Cell density was calculated as the number of cells (N_cells_) in the corresponding volume of the image sample (V_image_= 4.75 × 10^−2^ µL) multiplied by the number of images (N_images_), adjusted with the PF dilution coefficient (dil_PF_) as shown in Equation (4):

Cell density (cell mL^−1^) = N_cells_/(V_image_ × N_images_) × (dil_PF_) (4)

##### Circularity

The circularity quantifies the regularity of the outer surface of a particle and has been use to highlight changes in cell morphology due to contact with organic solvent in Gram-positive bacteria [43]. The circularity was calculated with the area (A) and perimeter (P) of the particle using Equation (5) and ranges between 0 and 1 [44].

Circularity = 4π × A/P^2^ (5)

Cells were classified into 21 classes of 0.05 width for the distribution analysis.

##### Cell Volume

Non-stressed *Dunaliella* cells exhibit a prolate spheroid shape whereas the shape of stressed cells varies from oblong to spherical [45]. Cells settle on their longitudinal section in the counting slide, allowing the major axis (b) to be measured as the maximum Feret diameter and the semi-minor axis (a) as the minimum Feret diameter. Individual cell volume was calculated using Equation (6).

Volume (µm^3^) = (π/6) × a × b^2^ (6)

Estimated cell volumes were classified into 27 classes of 200 µm^3^ width for distribution analysis. The biomass volume was calculated as the sum of all individual volumes.

##### Index of Potential Disruption

An index of potential cell disruption (IPCD) was calculated as the product of normalized cell volume (V_n_) and normalized cell circularity (C_n_) following Equation (9). The cell volume V_x_ was normalized between 0 and 1 with minimum and maximum values set to 0 and 2600 µm^3^, respectively (Equation (7)). The cell circularity C_x_ was normalized between 0 and 1 with minimum and maximum values set to 0.5 and 0.9, respectively (Equation (8)). Cells with volume or circularity outside of the respective ranges—which represented less than 10% of the population—were not counted.

V_n_ = (V_x_ − V_min_)/(V_max_ − V_min_) = (V_x_ − 0)/(2600 − 0) (7)

C_n_ = (C_x_ − C_min_)/(C_max_ − C_min_) = (C_x_ − 0.5)/(0.9 − 0.5) (8)

IPCD = V_n_ × C_n_ (9)

##### Biomass Integrity, Cell Disruption Yield and Rate

Biomass integrity (%) was calculated relative to the fresh biomass for cell density, biomass volume β-carotene concentration and dry weight (Equation (10)), where X_0_ is the initial amount and X_t_ is the amount after t seconds of extraction:

B_t_ (%) = 100 × X_t_/X_0_ (10)

Cell disruption yield (%) for each IPCD class was calculated following Equation (11) between the initial state (0 s) and the end of the treatment (240 s).

DY (%) = 100 × (X_0_ − X_240_)/X_0_ (11)

A first order model (Equation (12)) was used to calculate cell disruption rate (k_d_) for each volume class with X_0_ the initial cell concentration and X_t_ the concentration after the time Δt. Only value with R^2^ > 0.95 were plotted.

k_d_ (s^−1^) = −ln(X_t_/X_0_)/Δt (12)

#### 3.4.5. Cell Membrane Permeabilization

Cell permeabilization with Evans blue dye (EB) (Sigma-Aldrich, Saint Louis, MO, USA, >75%) was tested according to Hamer et al. (2002) [46]. To distinguish between irreversible and reversible permeabilization, EB (120 µL mL^−1^ of solution in the cell culture) was added in three different conditions: (A) without treatment, (B) before the treatment, (C) after each solvent treatment time with *n*-decane [47]. Thirty bright fields were represented in colour as explained in the Section Cell Density and the cell counter plug-in from Image J was used to manually count the cells which led the dye (bluish cells) and those which did not. The number of cells shown varied from 300 to 1200 cells per sample.

In agreement with Gateau et al. [8] cells can range into 5 categories, i.e., (1) the irreversibly permeabilized (IP), (2) the reversibly permeabilized (RP), (3) the naturally permeabilized (NAT-P), (4) the non-affected cells (NA) and (5) disrupted cells (DC), (Table 1). Thus, after mixing with solvent, a sample contained a certain proportion of each cell category. In addition, it is considered that each cell could exist under two states: non-permeabilized (non-stained) or permeabilized (stained). Therefore, one can write after a time of t seconds the Equations (13) and (14):

100% = IP_t_ (%) + RP_t_ (%) + NA_t_ (%) + DC_t_ (%) (13)

100% = Non-stained cells_t_ (%) + Stained cells_t_ (%) + DC_t_ (%) (14)

Because the number of cells is not fixed, disrupted cells percentage after t seconds of mixing with the solvent was calculated with the cell density after t seconds of mixing (C_t_) relative to initial cell density (C_0_) as follows in Equation (15):

DC_t_ (%) = 100·(C_0_ − C_t_)/C_0_ (15)

In the absence of mixing with solvent (A) all the cells remain by definition non-permeabilized, but only NAT-P cells will be stained with Evans blue as expressed in Equations (16) and (17):

Non-stained cells (%) = NA (%)(16)

Stained cells (%) = NAT-P (%)(17)

During mixing with the solvent, cells can turn from a non-permeabilized state to the permeabilized state resulting in the formation of IP and RP categories. Among the permeabilized cells, those that were able to reseal the plasma membrane from the RP category the other formed the IP one. When EB was added before mixing with solvent (B), only IP, RP and NAT-P cells should have been stained with EB. The categories of IP, RP and NAT-P cells cannot be distinguished individually but can be grouped together and defined as stained cells as expressed in Equations (18) and (19)

Non-stained cells (%) = NA (%)(18)

Stained cells (%) = NAT-P (%) + RP (%) + IP (%)(19)

When EB has been added after mixing with solvent (C), cells that resealed their membranes i.e., the RP should not be stained as expressed in Equations (20) and (21):

Non- stained cells (%) = NA (%) + RP (%)(20)

Stained cells (%) = NAT-P (%) + IP (%)(21)

By definition, NA and RP cells are alive after the treatment with *n*-decane. Their relative amounts can be combined to define the biocompatibility index (BI) which can be calculated using Equation (22):

BI_t_ (%) = NA_t_ (%) + RP_t_ (%) (22)

#### 3.4.6. Statistics and Plots

Extraction experiments were performed in triplicate. Error bars represent the standard deviation of the three replicates. Results were considered statistically different when the *p* value was under 0.05. It has been calculated using the software excel with ANOVA analysis. Boxplots were graphed on MATLAB, density scatter plots were made with Eilers and Goeman (2004) algorithm on MATLAB [48].

## 4. Conclusions

This study revealed that the culture conditions and the associated morphology (around 0.9 in circularity and cell volume below 600 µm^3^) of *D. salina* were important factors to be considered to drive the extraction of β-carotene with *n*-decane. The different culture conditions used highlighted the relationship between the disruption yield in a population, the cell volume, circularity and abundance of naturally permeabilized cells. New carotenoid production schemes can be envisioned by the growth of robust cells enriched in β-carotene cells under controlled pH, mixing and CO_2_ conditions to ensure biocompatible extraction. The behaviour of cells of different morphology at the liquid–liquid interface seems to govern both extraction efficiency and cell viability. Further, the ability of the cells to grow and reaccumulate β-carotene after one extraction step has to be studied before suggesting a biocompatible extraction process. Finally, the use of green solvents to extract β-carotene needs to be investigated as a benefit for future applications, including those related to medicine and pharmacy.

## Figures and Tables

**Table 1:** Cell response to the staining with Evans blue.

Cell Category	Abbreviation	EB Alone (A)	EB before Solvent (B)	EB after Solvent (C)
Non-affected	NA	−	−	−
Reversibly permeabilized	RP	−	+	−
Irreversibly permeabilized	IP	−	+	+
Naturally permeabilized	NAT-P	+	+	+
Disrupted cells	DC	/	/	/
